# The biogeochemical fate of nickel during microbial ISA degradation; implications for nuclear waste disposal

**DOI:** 10.1038/s41598-018-26963-8

**Published:** 2018-06-08

**Authors:** Gina Kuippers, Christopher Boothman, Heath Bagshaw, Michael Ward, Rebecca Beard, Nicholas Bryan, Jonathan R. Lloyd

**Affiliations:** 1 Research Centre for Radwaste Disposal & Williamson Research Centre for Molecular Environmental Science, School of Earth, Atmospheric and Environmental Sciences, University of Manchester, Oxford Road, Manchester, M13 9PL UK; 2 Leeds Electron Microscopy and Spectroscopy Centre, School of Chemical and Process Engineering, University of Leeds, Leeds, LS2 9JT UK; 3 Radioactive Waste Management Limited, Building 587, Curie Avenue, Harwell Oxford, Didcot, Oxfordshire OX11 0RH UK; 4 National Nuclear Laboratory Limited, Chadwick House, Warrington Road, Birchwood Park, Warrington, WA3 6AE UK; 5 Present Address: National Nuclear Laboratory Limited, Chadwick House, Warrington Road, Birchwood Park, Warrington, WA3 6AE UK

## Abstract

Intermediate level radioactive waste (ILW) generally contains a heterogeneous range of organic and inorganic materials, of which some are encapsulated in cement. Of particular concern are cellulosic waste items, which will chemically degrade under the conditions predicted during waste disposal, forming significant quantities of isosaccharinic acid (ISA), a strongly chelating ligand. ISA therefore has the potential to increase the mobility of a wide range of radionuclides via complex formation, including Ni-63 and Ni-59. Although ISA is known to be metabolized by anaerobic microorganisms, the biodegradation of metal-ISA complexes remains unexplored. This study investigates the fate of a Ni-ISA complex in Fe(III)-reducing enrichment cultures at neutral pH, representative of a microbial community in the subsurface. After initial sorption of Ni onto Fe(III)oxyhydroxides, microbial ISA biodegradation resulted in >90% removal of the remaining Ni from solution when present at 0.1 mM, whereas higher concentrations of Ni proved toxic. The microbial consortium associated with ISA degradation was dominated by close relatives to *Clostridia* and *Geobacter* species. Nickel was preferentially immobilized with trace amounts of biogenic amorphous iron sulfides. This study highlights the potential for microbial activity to help remove chelating agents and radionuclides from the groundwater in the subsurface geosphere surrounding a geodisposal facility.

## Introduction

The policy of the UK Government (and those of other nuclear nations) is to dispose of long-lived intermediate level waste (ILW) via engineered deep underground geological disposal facilities (GDFs)^1^. Post-closure, a cementitious GDF will become saturated with groundwater, which through interaction with the cementitious materials that make up the engineered barrier components, will result in the development of a highly alkaline plume. These high pH conditions are expected to minimize the mobility of toxic or hazardous radionuclides and inhibit most microbiological processes. However, organic ligands have the potential to impact on the solubility of radionuclides in these wastes. Ligands can be either present in ILW, for example the decontamination agents ethylenediaminetetraaceteic acid (EDTA) or nitriloaceteic acid (NTA), or may form under GDF conditions from the chemical degradation of organic materials. Of particular interest are cellulosic materials, which are present at high loadings and have been shown to undergo alkaline hydrolysis at elevated pH and calcium concentrations, resulting in the production of small organic acids in the pore fluids^2^. Under GDF conditions, the main stable end product is expected to be isosaccharinic acid (ISA)^3–8^. Even though ISA is known to sorb to cement^9,10^, it can form strong water-soluble complexes with some priority radionuclides, especially divalent cations, such as nickel (Ni)^9,11^. Nickel is chiefly valuable in Ni-based steel alloys to minimize corrosion of steel^12^. In a reactor, stable Ni that is part of nuclear reactor steel components can be irradiated from the fuel and form Ni-63 (half-life 9.9 × 10^1^ years) and Ni-59 (half-life 7.6 × 10^4^ years) as activation products^13,14^. Upon groundwater resaturation of the GDF, steel corrosion will release Ni, which should be poorly soluble in the alkaline porewaters (pH 11.5 to 13.5) and sorb readily onto cement^9,15^. However, the formation of aqueous complexes between ISA and Ni (at a 2:1 stoichiometry)^16^ may significantly enhance the transport of Ni in the groundwater and their fate upon disposal needs to be considered as part of the safety assessment of a GDF. Thus, there is concern regarding the fate of Ni, alongside other priority radionuclides and metals that may complex with ISA, in and around a cementitious GDF. Nickel was chosen for this study as a model radionuclide, from which conclusions on the fate of other cationic radionuclides forming complexes with ISA can be drawn.

Previous studies on metal-microbe interactions have focused on a range of microorganisms, including anaerobic metal- and sulfate-reducing bacteria, for their ability to immobilize radionuclides via mechanisms including bio-reduction, bioaccumulation, biosorption or biomineralisation^17–24^. To this date, studies on Ni removal relevant to nuclear disposal have looked at Ni intercalation into biominerals, such as hydrogen uranyl-phosphates^25–27^, and the biodegradation of organic ligands with subsequent Ni precipitation, such as Ni-citrate complexes^18,28,29^.

Microbially-mediated processes have been shown to control the fate of ISA, which can be used as an organic carbon source and electron donor for microbial metabolism, under a range of biogeochemical conditions, including nitrate and Fe(III) reduction at high pH^30–33^ and neutral pH^34,35^. However, no studies to date have addressed the fate of radionuclide-ISA complexes, such as of Ni-ISA, which could potentially form under repository conditions, and have the potential to influence the properties of components in a GDF^16,36^. Furthermore biodegradation of Ni-ISA, could lead to sorption of the radionuclide, or its incorporation into new biominerals and sorption surfaces. Other scavengers for metals and radionuclides, could be Fe(III) oxyhydroxides present in a GDF from the corrosion of steel under aerobic conditions in the “open phase of a GDF”, or naturally occurring Fe(III)-bearing minerals in the wider circumneutral pH geosphere (or “far field”). These Fe(III)-bearing minerals may further play a role as alternative electron acceptors, if bioavailable, sustaining ISA metabolizing anaerobes in and around a GDF. Such Fe(III)-bearing minerals could release Ni upon reduction to Fe(II)^37,38^ and need to be considered in biodegradation studies. Other potentially relevant electron acceptors in the far field could include Mn(IV) minerals or sulfate, while nitrate could be sourced from waste materials or far field groundwaters, supporting ISA oxidation. The microbial reduction of Fe(III)-minerals in the subsurface and associated secondary mineral transformations could therefore have a profound influence on the fate of Ni and other priority metals and radionuclides in the geosphere. In this study, we have used a microbial inoculum obtained from an alkaline, Ca^2+^-rich lime kiln site, which is an analogue for a cementitious GDF and known to contain ISA-degrading microorganisms^30,34^. This inoculum was used to investigate the fate of Ni during ISA biodegradation under Fe(III)-reducing conditions possibly relevant to the wider geosphere of a GDF, including identifying the role of key Fe(II)-bearing biominerals in Ni immobilization.

## Results

### Nickel in solution

In this study the fate of non-radioactive Ni was explored in ISA-degrading, Fe(III)-reducing microbial cultures. Prior to the study, abiotic factors in the system that could control the solubility of Ni were considered, e.g. the presence of Fe(III)oxyhydroxide that exhibits high adsorptive capacities for metal ions^39^. The solubility of Ni (at 0.1 mM Ni and 1 mM Ni) was tested in freshwater minimal medium (FWM) at neutral pH, containing (i) medium only, (ii) medium plus ISA, (iii) medium plus Fe(III) or (iv) medium plus ISA and Fe(III) (Fig. S1). Biosorption of Ni onto cells was not specifically tested, as it is considered to be low^40,41^. In the presence of the medium only, approximately 90–91% Ni remained in solution, whilst in the presence of ISA the solubility of Ni was increased to approximately 100%, regardless of the initial Ni concentration. In the medium that contained Fe(III)oxyhydroxides added as a solid, the solubility of Ni dropped to around 64% at 0.1 mM Ni and to 15% at 1 mM Ni, but was increased by approximately 10% in the medium containing Fe(III) and ISA at 1 mM Ni, but remained unchanged at 0.1 mM Ni.

These initial tests showed that Ni had a great solubility in the medium which was enhanced by ISA, and therefore has the potential to increase radionuclide mobility in groundwater systems. However, the presence of competing Fe(III)oxyhydroxides decreased the solubility of the Ni.

### Development of an Ni-ISA biodegrading, Fe(III)-reducing enrichment experiment

#### Biogeochemistry

**Figure 1:**
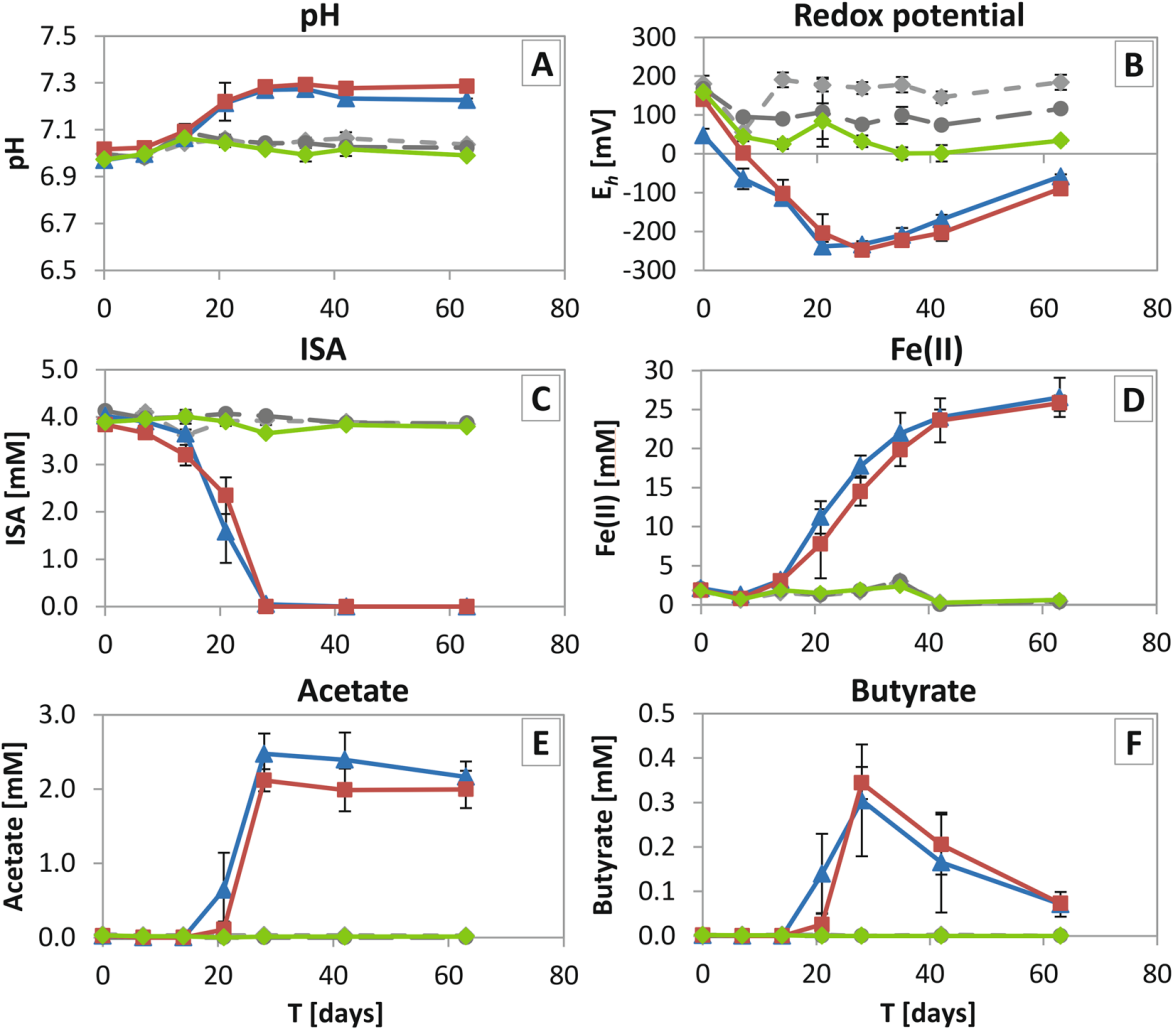
Geochemical information of Ni-ISA biodegrading, Fe(III)-reducing enrichment experiment. Panels describe the following summarized for all microcosms: (**A**) pH; (**B**) *E*_h_; (**C**) ISA concentration; (**D**) Fe(II) ingrowth; (**E**) acetate and (**F**) butyrate. Symbols are: sterile microbial inoculum (autoclaved) with 1 mM Ni (grey diamonds; hidden by the “no microbial inoculum” control), “no microbial inoculum” with 1 mM Ni (dark grey circles), and incubations with microbial inoculum and with either 0 mM Ni (blue triangles), 0.1 mM Ni (red squares), or 1 mM Ni (green diamonds).

An anaerobic enrichment culture representative of Fe(III)-reducing bacteria, possibly present in the geosphere surrounding a GDF, was developed using ISA complexed with Ni as the electron donor and Fe(III)oxyhydroxide as the electron acceptor. Microbial inocula were obtained from a lime workings site, with high calcium concentrations and alkaline pH values, and were enriched to stable cultures with ISA as the sole carbon source at circumneutral pH. The microbially active incubations contained either no nickel, 0.1 mM Ni or 1 mM Ni. Medium with microbial enrichments without Ni or with 0.1 mM Ni concentration, there was an increase in pH from 7.0 to 7.3 while the *E*_h_ fell to around −250 mV after approximately 14 days (Fig. 1A,B). This coincided with a drop in ISA levels, which was no longer detected after approximately 28 days (Fig. 1C), while volatile fatty acids (VFAs), comprising acetate and butyrate accumulated and peaked at 28 days (Fig. 1E,F). Thereafter, acetate remained stable in solution during an extended incubation time (62 days), whilst butyrate concentrations decreased after ISA was depleted. Fe(III) reduction, monitored using the ferrozine assay by Fe(II) ingrowth (Fig. 1D), was also initiated during the period of ISA biodegradation, and continued after ISA was depleted until day 42, after which it started to plateau at approximately 26 mmoles L^−1^ Fe(II). It should be noted that the medium also contained comparatively low levels of sulfate (0.13 mM), which decreased in the presence of 0.1 mM Ni and without Ni, alongside ISA removal, until it was fully depleted at day 42 (Fig. S2). In contrast, in the enrichment cultures supplemented with 1 mM Ni, data for pH, *E*_h_ and concentrations of ISA, Fe(II), and sulfate all remained stable, and Fe(III) and sulfate were not reduced. Abiotic influences, two control experiments were set up, containing ISA and Fe(III)oxyhydroxide, either with an autoclaved inoculum or without the inoculum added. ISA concentration remained stable in these controls (Fig. 1B), while the pH remained unchanged, the *E*_h_ showed only a minor drop over the incubation time (Fig. 1A,D) and Fe(II) levels remained constant, all consistent with abiotic influences playing a negligible role in these experiments (Fig. 1E).

#### Fate of nickel

**Figure 2:**
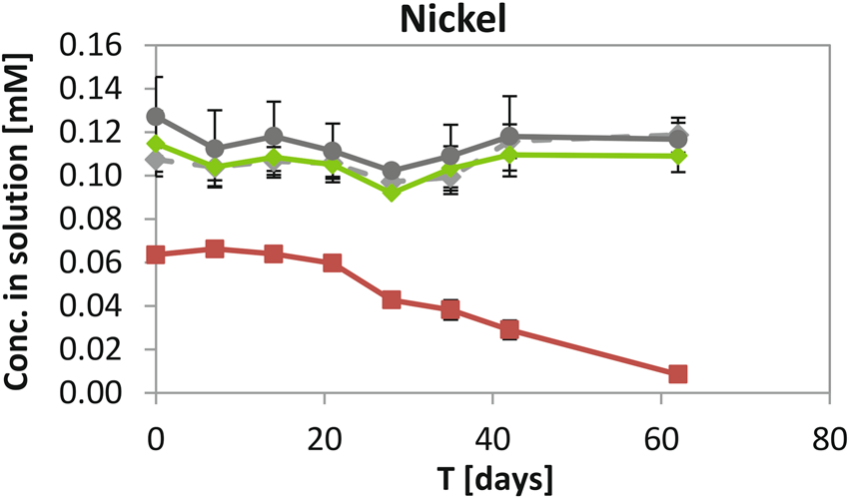
ICP-AES analysis of Ni in solution [mM] in the Ni-ISA biodegrading, Fe(III)-reducing enrichment experiment. All bottles contained 4 mM ISA and 25 mM Fe(III) oxyhydroxide with a sterile (autoclaved) microbial inoculum and 1 mM Ni (grey diamonds), or no microbial inoculum, but 1 mM Ni (dark grey circles), or with microbially active inoculum and either 0.1 mM Ni (red squares), or 1 mM Ni (green diamonds).

The concentrations of Ni in the supernatants of these experiments were also monitored by ICP-AES, to help identify the fate of the metal during ISA biodegradation and Fe(III) reduction (Fig. 2). At the start of incubation, soluble Ni concentrations were 64% (64 µM) of the 0.1 mM Ni added, and 12% (115 µM) of the 1 mM Ni added to the microbially-active cultures. These results were in agreement with data from the initial Ni solubility tests and were most likely a result of sorption to the surface of the Fe(III)oxyhydroxides that were added.

After partial precipitation of the Ni (about 90%) in the control incubations (with either autoclaved inoculum or no added inoculum), the remaining soluble Ni concentrations remained stable, confirming that sorption equilibrium or abiotic precipitation were not disturbed under these experimental conditions. Similarly, after initial sorption of about 90%, no decrease of Ni concentrations in solution was observed in the microbially active enrichment culture containing 1 mM Ni, where ISA degradation was absent. In the microbially active 0.1 mM Ni incubation, the Ni concentration in solution was also reduced at the start of the incubation to about 64% of Ni added (presumably due to sorption onto any residual insoluble Fe(III)). However in contrast to the treatments which showed a lack of ISA degradation, the Ni concentration started to decrease from day 14 from 64 µM to 8.5 µM (about 9%) final concentration in solution at the end of the incubation (Fig. 2), and remained at this value throughout an extended incubation time of 200 days (data not shown). Thus, following Ni sorption onto Fe(III)oxyhydroxides (36% removal), the oxidation of ISA and reduction of Fe(III) and sulfate was concomitant with the almost complete removal (90%) of the Ni added.

#### Molecular Ecology

**Figure 3:**
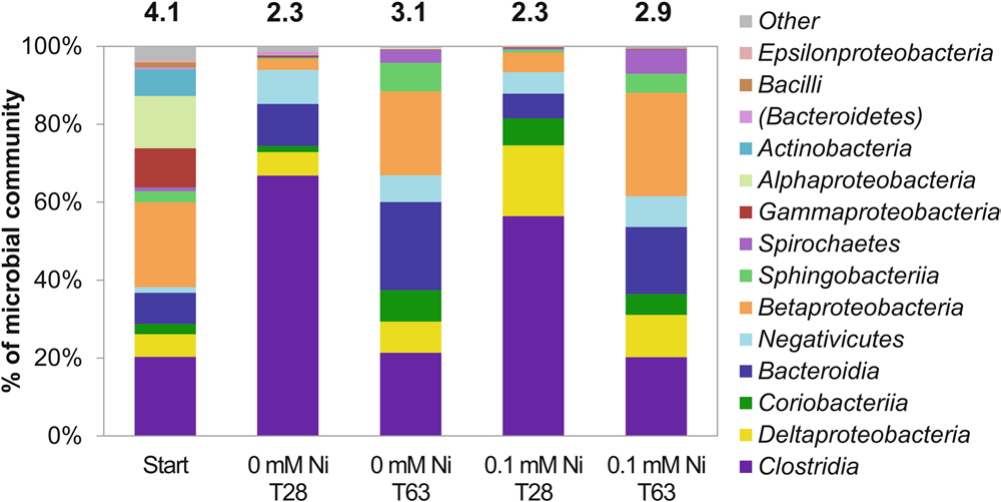
16S rRNA gene sequencing data from the Ni-ISA biodegrading, Fe(III)-reducing enrichment experiment. Diagram shows phylogenetic Classes at selected time points. Notation of Shannon H biodiversity index on top of columns as a measure of microbial community diversity.

A stable microbial community was enriched from lime kiln sediment samples over seven consecutive transfers in ISA/Fe(III)-containing medium, with the last transfer used in the Ni-ISA biostimulation experiments discussed above. DNA was extracted from these final cultures at selected time points and the 16S rRNA genes present were amplified and sequenced, to identify changes in microbial community composition that may have controlled the fate of the Fe and Ni. The chosen profiles were from the 0 mM Ni and 0.1 mM Ni experiments at 0 hours, at 28 days, the point at which ISA was depleted, and at 63 days, the point where Fe(III) reduction levelled off (Fig. 3). α-rarefaction curves showed a dramatic decrease in microbial diversity from the original sediment inoculum (approximately 600 distinct sequences detected)^21^ to the seventh transfer of the enrichments (approximately 190 distinct sequences detected at the start of this experiment; Fig. S3). A further modest decrease in microbial diversity was noted over the duration of this experiment, whereby the 0 mM Ni and 0.1 mM Ni supplemented enrichments contained approximately 160 distinct sequences. The decrease in community microbial diversity was confirmed by calculating the Shannon H indices (values are shown above the columns in Fig. 3).

Analysis of 16S rRNA gene sequences obtained from the 0.1 mM Ni experiment after ISA depletion (28 days) showed enrichment in organisms most closely affiliated with the Gram-positive Class *Clostridia*, representing approximately 56% of the total abundance of bacteria detected. This Class comprised mainly of members of the family *Ruminococcaceae* (53% of genes detected), known to possess a multi-enzyme cellulosome complex that could play a central role in carbohydrate metabolism^42^. Another important Class detected was the Gram-negative *Deltaproteobacteria* comprising approximately 18% of sequences detected. This Class was dominated by sequences affiliated most closely (97% to 99% match) with the Fe(III)-reducing family *Geobacteraceae* (16% of sequences detected). Despite representing less than 2% of the sequences, detection of members of the *Desulfovibrionaceae* was considered potentially important, as these organisms were affiliated with the sulfate-reducing bacteria *Desulfovibrio putealis B7–43* (1% of sequences, 98% match) and *Desulfovibrio L7* sp. (0.65% of sequences, 100% match). A second 16S rRNA gene profile was obtained from the 0.1 mM Ni experiment when Fe(III) reduction had finished (day 63). This had a higher percentage of sequences affiliated with Betaproteobacteria (26% of sequences), of which most of the sequences (25%) were related to *Dechlorosoma suillum PS* (100% match) from the genus *Azospira*, an anaerobic, perchlorate-reducing organism^43^. The second most abundant Class detected was again most closely affiliated with *Clostridia* (20% of sequences) followed by the Bacteroidia (17% of sequences), of which most sequences were affiliated with an uncultured bacterium from the vadinBC27 wastewater-sludge group (11% of sequences), from the genus *Rikenellaceae*, which are described as anaerobic, mesophilic carbohydrate-fermenting organisms^44^.

#### Biomineralogy

To help define the fate of Ni in the ISA-degrading, Fe(III)-reducing cultures, the mineralogical end-products in these experiments were assessed using X-ray Diffraction (XRD), Transmission Electron Microscopy (TEM) and Environmental Scanning Electron Microscopy (ESEM).

At the start of the Ni-ISA biostimulation experiment, a brick-red precipitate was observed, which is likely to be ferrihydrite as detected in XRD analyses (Fig. S4). This starting material remained unchanged in the sterile and no electron donor controls, and also the incubations with 1 mM Ni added. This mineral phase was converted to a dark grey precipitate where ISA metabolism was observed (microbially active experiment without Ni and with 0.1 mM Ni). TEM analysis of the red precipitate in the sterile control showed the presence of fine grained material, while selected area electron diffraction (SAED) revealed two broad rings at about 1.49 Å and 2.5 Å, all consistent with 2-line ferrihydrite that is of irregular, poorly ordered nanoparticulate dimensions (2–4 nm)^45–48^. The XRD patterns (Fig. S4) obtained for the bio-reduced samples in the absence of Ni and with 0.1 mM Ni added, revealed two sharp peaks at 2Ɵ = 13.2° and at 2Ɵ = 31.9°, corresponding to siderite (FeCO_3_) and vivianite Fe_3_(PO_4_)_2_·8H_2_O (Fig. S4). The relative abundance of vivianite:siderite was very similar at both ISA-degrading treatments of about 40%:60% in the absence of Ni and 35%:65% at 0.1 mM Ni, respectively. Fe(II) in the siderite structure was partially replaced by Ca(II), as previously noted (Kuippers *et al*., Submitted 2017).

**Figure 4:**
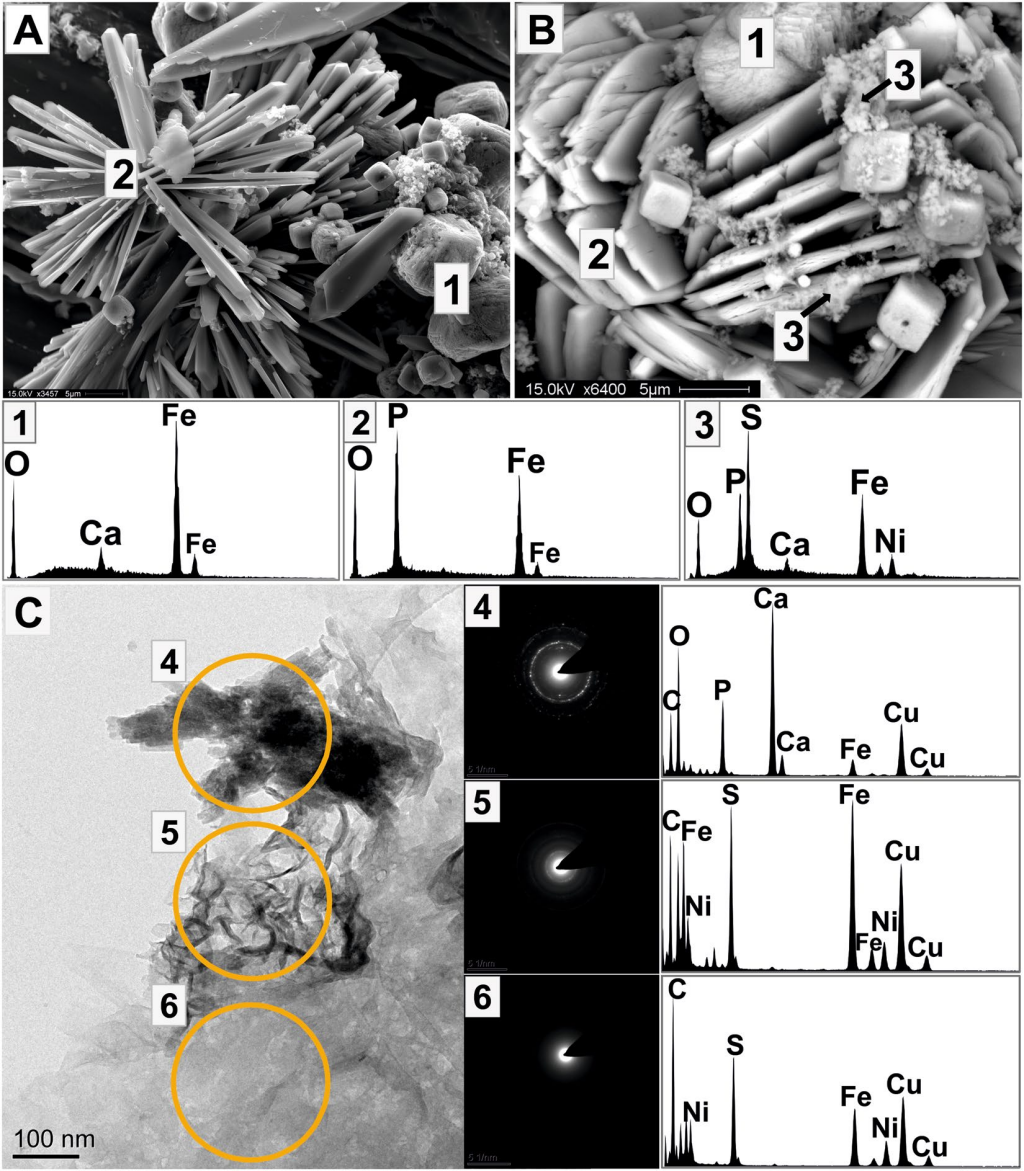
ESEM (**A**,**B**) and TEM (**C**) images of biominerals from the Ni-ISA biodegrading, Fe(III)-reducing enrichment experiment with 0.1 mM Ni with representative EDS profiles. (**A**) and (**B**): Biominerals identified were (1) siderite, (2) vivianite and (3) an amorphous phase, comprising S, Fe, and Ni. C: (4) a crystalline area without Ni and (5, 6) weakly crystalline areas with Fe, S and Ni.

ESEM imaging of the post-reduction precipitates (with 0.1 mM Ni; Fig. 4A,B), confirmed the identity of (1) siderite and (2) vivianite crystals, which did not show Ni in the corresponding EDS profile. Precipitates of less crystalline morphology were also noted, comprising relatively high amounts of Fe, S and Ni, (EDS 3 in Fig. 4B); peaks of P, Ca and O were probably background signal from the underlying vivianite crystal (EDS 1 and 2 in Fig. 4A). Similarly, in TEM images, distinct crystals of angular shape that contained Ca, Fe, P and O (60–70 nm length; 12–16 nm diameter; area 4 in Fig. 4C) had matching lattice *d* spacings for siderite in the corresponding diffraction pattern: 2.72 (104), 2.17 (113), 1.47 (122), 1.41 (214) and 1.27 Å (0210)^49,50^, but did not contain Ni. In contrast, a weakly crystalline area (area 5 in Fig. 4C), deduced from very faint rings in the diffraction pattern with *d* spacings at 5.18 Å, 2.59 Å, 1.78 Å and 1.68 Å in the diffraction pattern, had high peaks of S and Ni, and some contributions of Fe, consistent with literature values for nanocrystalline mackinawite^51–55^. Another area was amorphous, with no rings in the diffraction pattern, but consisted of mainly Fe, S and Ni (area 6 in Fig. 4C). Finally, TEM mapping suggested close association of Ni and S areas, whilst P, Ca and O were accumulated in separate locations, and Fe was detected throughout the mapped area (Fig. S5). Additional PHREEQC modelling was carried out to estimate the Ni solubility. Results from the modelling suggested that Ni was undersaturated in solution under anoxic conditions and before reduction (Fig. S6) while after bio-reduction precipitation of millerite and mackinawite was predicted (Fig. S7).

## Discussion

Ni is an important constituent in steel alloys used for nuclear reactor components. Irradiation of stable Ni leads to the formation of Ni-63 and Ni-59 activation products, hence there is great interest in understanding the fate of Ni during the (bio)geochemical evolution of a GDF and the surrounding geological environment.

Here we studied the fate of a Ni-ISA complex in circumneutral enrichment cultures containing Fe(III) as the main terminal electron acceptor. It should be noted, however, that to facilitate mineralogical investigations that underpin this study, significantly higher concentrations of Ni were added, than those expected in a GDF environment for ILW, which will range around 3.8 * 10^−7^ M within 10^6^ years of GDF evolution^9^. In these incubations, ISA biodegradation coincided with Fe(III) reduction, and led to a significant decrease in the concentration of Ni when supplied at 0.1 mM. At 1 mM Ni concentration, no ISA removal occurred, highlighting the strongly inhibitory impact of higher Ni concentrations on microorganisms. In the microbially active experiment at 0.1 mM Ni, fermentation played a significant role as approximately 60% of the carbon from ISA degradation was recovered as acetate and butyrate. Other end-products identified included the crystalline Fe(II) biominerals, siderite (Fe(II)-carbonate) and vivianite (Fe(II)-phosphate). Although ferrihydrite is considered highly bioavailable to Fe(III)-reducing bacteria^56^, the production of fully reduced Fe(II) minerals from this substrate, rather than a mixed Fe(II)/Fe(III) valence iron oxide, such as magnetite, is not commonly observed in laboratory studies^57,58^, but has been previously noted when ISA was supplied as an electron donor^21^. Chelating ligands are known to enhance microbial Fe(III) reduction rates^59–62^, and since ISA has been shown to complex with Fe(III) minerals^8^, ISA may have increased the solubility and hence bioavailability of ferrihydrite to microbial Fe(III) reduction in our experiments. We conclude therefore, that chelation of ISA with Fe(III) will result in the production of Fe(II) biominerals not normally observed in natural environments.

The negative standard reduction potential of the Ni(II)/Ni(0) couple (E0 = −0.26 V)^63^ makes Ni highly resistant to microbially-mediated reduction under pH-neutral conditions (Tsezos *et al*.^41^). Therefore, capture of Ni via interaction with bio-mineralogical products is a far more feasible endpoint under anoxic ISA-degrading conditions. In these Fe(III)-reducing experiments, the endpoint minerals included an Fe(II)-carbonate phase (siderite) and Fe(II)-phosphate phase (vivianite). Siderite is known to play a role in trace metal immobilization because cations of similar size to Fe(II), such as Co(II), Cr(III), Ni(II), can be readily incorporated into the crystal structure in place of ferrous iron^64,65^. In these experiments only Ca(II) was incorporated into the siderite structure upon metabolism of Ca(ISA)_2_, whereas Ni(II) was not found to be associated with the biomineral, nor was Ni associated with vivianite. However it should be noted that Fe(II)-bearing minerals, including siderite and vivianite, are known to mediate the reduction of soluble Tc(VII)^62,66,67^ or Np(V)^68^ to insoluble tetravalent forms. Additionally, vivianite has also been shown to reduce soluble U(VI) to immobile U(IV)^69^. Thus, siderite and vivianite may decrease the mobility of this and other priority radionuclides in the wider geosphere.

Regarding the fate of Ni, EDS profiles from TEM and ESEM images showed that Ni accumulated together with Fe and S in amorphous precipitates, consistent with mackinawite (FeS), although this mineral could not be detected with XRD. Mackinawite is a common metastable iron-sulfide mineral in anoxic sediments and a possible precursor to greigite and pyrite formation^52,70–72^. Furthermore it is known to substitute Ni for Fe, into the sulfide mineral structure^51,73–75^. The identification of Ni-containing iron-sulfide phases in the ISA bio-stimulated experiment, accompanied by an enrichment of *Desulfovibrio* sp., is consistent with the production of sulfide by sulfate-reducing bacteria that can act as a precursor to precipitate Ni. This is in agreement with PHREEQC modelling that suggested formation of mackinawite (FeS) and millerite (NiS) in these experiments. The PHREEQC database does not include data for mixed phases, such as Fe_(1-x)_,Ni_x_S, and so it predicts the formation of these separate phases. However, since pure Ni-sulfides, such as heazelwoodite (Ni_3_S_2_) or millerite (NiS), are generally only observed in systems that are low in Fe^75^, and (Fe,Ni)S has a greater stability over pure FeS^70,73,74^, the modelling results can be interpreted as formation of a mixed Fe_(1-x)_,Ni_x_S phase, which is in agreement with TEM and ESEM imaging, following the equation:

1 $$(1-{\rm{x}}){{\rm{Fe}}}_{({\rm{aq}})}^{2+}+{{\rm{xNi}}}_{({\rm{aq}})}^{2+}+{{\rm{H}}}_{2}{{\rm{S}}}_{({\rm{aq}})}\to {{\rm{Fe}}}_{(1-{\rm{x}})},{{\rm{Ni}}}_{{\rm{x}}}{{\rm{S}}}_{({\rm{s}})}+2{{\rm{H}}}_{({\rm{aq}})}^{+}$$

The immobilization of Ni precipitated as a mixed (Fe,Ni)S phase is thus a result of combined microbial activity by Fe(III)- and sulfate-reducing bacteria.

16S rRNA gene profiling of the enrichment identified key soil microorganisms that may have been involved in the degradation of ISA and thus supported Ni immobilization. The 16S rRNA gene profiles of the 0.1 mM Ni and without Ni experiments were very similar, being dominated by fermenting organisms associated with members from the family *Clostridia* during ISA degradation (day 28), whilst *Betaproteobacteria* increased in later stages of the incubation, when Fe(III) reduction became more important (day 63). Although close relatives to well-known Fe(III)-reducing bacteria such as *Geobacter* species were detected in these cultures, we cannot discount the involvement of other more abundant organisms in mediating this process. Finally, although Ni is an essential trace element for several enzymes^76–79^, at higher concentrations it poses a toxic threat to microorganisms^80,81^. Such an inhibitory effect of Ni was observed at 1 mM concentration, where no ISA biodegradation (or Fe(III) reduction) was recorded.

In summary, it was shown that the solubility of Ni in ISA-degrading Fe(III)-reducing enrichment cultures was influenced by two dominant factors; the mobilization of Ni by complexation with ISA, and the competing immobilization of Ni by sorption to Fe(III)oxyhydroxides. The overall fate of Ni in the system was defined by microbial metabolism, which was driven by biodegradation of ISA. The combined activity of Fe(III)- and sulfate-reducing microorganisms resulted in >90% Ni incorporation into iron-sulfides, presenting a stable sink for the Ni. Given the high abundance of sulfate as electron acceptor in deep subsurface groundwaters in the UK^82^ and elsewhere, the fate of Ni and other radionuclides in the deep geosphere surrounding a GDF, is likely to be governed by sulfidation reactions. These processes will be potentially mediated by Fe(III)- and sulfate-reducing bacteria in the deep subsurface that have been stimulated by the release of organics such as ISA, that may migrate in the alkaline groundwater plume from a cementitious GDF. Since ISA oxidation coupled to Fe(III) and sulfate reduction is energetically unfavorable in the alkaline GDF “near field”^83^, precipitation of Ni and other radionuclides is expected to occur in the geological “far field” of a GDF and in low pH niches of the “near field”. It should be noted that also direct abiotic interactions within the geosphere are possible once the strong complexant ISA has been biodegraded, e.g. with clay minerals, known to adsorb cationic Ni due to their negatively charged surfaces, and thus are able to retard the movement of Ni^77,84,85^. Additionally to the trace levels of FeS formed, ISA degradation in our experiments supported dissimilatory Fe(III) reduction that produced the Fe(II) minerals siderite and vivianite. These Fe(II) minerals of are known to form at moderately high pH^86^, and to abiotically mediate the reduction of priority radionuclides to insoluble forms. Thus, it is likely that microbial metabolism in the deep subsurface biosphere surrounding a GDF will play a significant role in minimizing the transport of mobile radionuclide-ISA complexes, through the fermentation of ISA at high pH^31^, and further coupling of biodegradation reactions to the reduction of electron acceptors such as Fe(III) and sulfate in and around the GDF, extending to the circumneutral deep subsurface biosphere. Consequently it is likely that a “bio-barrier” will develop, which could support the containment of priority radionuclides, including Ni-63 and Ni-59, in addition to other physical and chemical barriers. Clearly further research is warranted in this area, including studies to define the impact of microbial metabolism on ISA complexed to other radionuclides, including Am(III), Th(IV) and U(VI). There exists also the need to focus on more realistic subsurface *in situ* conditions to better quantify the efficiency of metal and radionuclide immobilization upon ISA degradation. Although it is clearly a significant challenge to quantify the impact of microbial metabolism in GDF scenarios, over long time periods within highly complex biogeochemical gradients, it is reasonable to expect that such studies would support a reduction in conservatisms in future GDF safety cases.

## Methods

### Experimental conditions

#### Fresh water minimal (FWM) medium preparation

FWM was prepared in deionized water and contained 30 mM NaHCO_3_, 4.7 mM NH_4_Cl, 4.4 mM NaH_2_PO_4_·H_2_O, 1.3 mM KCl, and 0.3 mL of mineral and vitamin stock solutions^87^.

#### α-Ca(ISA)_2_ preparation

α-lactose monohydrate and Ca(OH)_2_ was used for preparation of Ca(ISA)_2_ following the protocol of^88^.

#### Fe(III)oxyhydroxides preparation

0.6 M FeCl_3_ were hydrolyzed in six washing steps with 18Ω de-ionized water (DIW), whilst the pH was continually adjusted under stirring to 7 by addition of 10 M NaOH solution. With the ferrozine assay the final Fe(III) concentration of the resultant precipitate was determined.

#### Nickel stock preparation

Stock solutions of 0.1 M and 0.01 M NiCl_2_·6H_2_O (n = 237.7 g/mol) were prepared with deionized water and filter-sterilized prior to use (0.22 µm, diam. 33 mm, Millex-GP, Sigma-Aldrich).

#### Nickel solubility

To help determine the impact of the growth medium on Ni solubility, Ni was added at 0.1 mM Ni or 1 mM Ni to 100 mL serum bottles, containing 30 mL (i) FWM, (ii) FWM plus 20 mmoles L^−1^ Fe(III), (iii) FWM plus 4 mM ISA, or (iv) FWM plus 20 mmoles L^−1^ Fe(III) plus 4 mM ISA. The solutions were incubated in the dark at 20 °C for 7 days and then sampled for analysis by ICP-AES.

#### Ni-ISA biodegrading, Fe(III)-reducing enrichment experiment

Anaerobic Fe(III)-reducing microbial communities were enriched from shallow subsurface sediments that were collected from a site contaminated by legacy lime workings at Harpur Hill, in Buxton, Derbyshire, UK^34^. Cultures were prepared with a 1% (v/v) inoculum added to 100 mL serum bottles containing 30 mL FWM. ISA (as Ca(ISA)_2_) was added to 4 mM as sole carbon source and 25 mmoles L^−2^ of poorly soluble Fe(III)oxyhydroxide was added as the terminal electron acceptor (TEA) to support anaerobic growth. The bottles were sealed with butyl stoppers and flushed with a N_2_/CO_2_ (80:20) gas mixture for 5 min to provide anaerobic conditions and to adjust a pH of 7 and incubated in the dark at 20 °C. After Fe(III) reduction had reached a plateau, a 1% vol/vol inoculum was transferred to fresh medium. For this study, the seventh consecutive transfer was used to prepare five tests in the medium as above in triplicate: to three incubations containing a microbially active inoculum, ISA and Fe(III)oxyhydroxide, nickel was added at the following concentrations: (i) 0 mM Ni, (ii) 0.1 mM Ni, or (iii) 1 mM Ni, alongside (iv) a sterile microbial inoculum (autoclaved), containing ISA, Fe(III)oxyhydroxide and 1 mM Ni and (v) an abiotic control without microbial inoculum but with ISA, Fe(III)oxyhydroxide and 1 mM Ni were created. Time-based samples were collected aseptically and frozen immediately at −20 °C until further analysis.

### Geochemical analyses

The pH was monitored throughout the experiment with a calibrated Mettler Toledo FEP20 digital meter equipped with a Fisherbrand FB68801 electrode and the redox potential with a Denver Instrument Accumet digital meter equipped with a Mettler Toledo Inlab Redox Micro ORP. Microbial Fe(III) reduction was monitored by the ferrozine spectrophotometric assay quantifying Fe(II) following the protocol from Stookey^89^. Total bioavailable Fe was determined at the beginning and end of the experiment by digesting 20 µL of sample in 980 µL of 0.5 N HCl and 0.25 N hydroxylamine-HCl, and biogenic Fe(II) was determined by digestion of 20 µL of sample in 980 µL of 0.5 N HCl, followed by the ferrozine assay^90^.

#### Ion-Exchange Chromatography

ISA and organic acids were analyzed using ion exchange high performance liquid chromatography (IE-HPLC), using a Dionex ICS5000 Dual Channel on Chromatograph, fitted with a Dionex AS-AP auto sampler that was connected to a CD20 conductivity detector. The columns were a Dionex Capillary (50 × 0.4 mm) AG11-HC 4 µm guard column and Dionex Capillary (250 × 0.4 mm) AS11-HC 4 µm analytical column. A Dionex ACES300 Chemical Suppressor was used for background reduction. The mobile phase was concentrated KOH mixed with high purity water at a flow rate of 0.015 mL/min. Background suppression was hold at 13 mA.

#### Inductively Coupled Plasma Atomic Emission Spectrometer (ICP-AES)

ICP-AES was used to quantify total Ni in solution. Samples were prepared by centrifugation to remove any suspended particles and the supernatants were diluted to below <10 ppm Ni in 2% nitric acid. Samples were analyzed together with standards (from 10 ppm stocks obtained from VWR) on a Perkin-Elmer Optima 5300 DV.

### Microbial analyses

#### 16S rRNA gene sequencing

16S rRNA gene sequencing was performed with the Illumina MiSeq platform (Illumina, San Diego, CA, USA) using a Roche ‘Fast Start High Fidelity PCR System’ (Roche Diagnostics Ltd, Burgess Hill, UK). The primers used were 515 F (5′-GTG YCA GCM GCC GCG GTA A-3′) and 806 R (5′-GGA CTA CHV GGG TWT CTA AT-3′), targeting the V4 hyper variable regions for 2 × 150-bp paired-end sequencing^91,92^. PCR was performed in 50 µL reactions, starting with the denaturation at 95 °C for 2 min, followed by 36 cycles of 95 °C for 30 s, 55 °C for 30 s, 72 °C for 1 min, and a final extension step of 5 min at 72 °C. PCR products were cleaned up and normalized to ~20 ng each using the SequalPrep Normalisation Kit (Fisher Scientific, Loughborough, UK). PCR amplicons were pooled in equimolar ratios, using a 4 pM sample library spiked with 4 pM PhiX, to a final concentration of 10%^93^. Sequence analysis was performed using an Agilent BioAnalyzer at the Centre for Musculoskeletal Research, Manchester^93^. Raw sequences were divided into samples by barcodes (up to one mismatch was permitted) using a sequencing pipeline. Quality control and trimming was performed using Cutadapt^94^, FastQC^95^, and Sickle^96^. MiSeq error correction was performed using SPADes^97^. Forward and reverse reads were assembled to full-length sequences with PANDAseq^98^. Using ChimeraSlayer the sequences were screened for chimeras which were removed subsequently^99^. OTU’s were generated with UPARSE^100^ and classified by USEARCH^101^ at the 97% similarity level, and singletons were removed. The taxonomic assignment was performed by the RDP classifier^102^ and α-rarefaction curves were generated in Qiime^103^. The raw sequencing data have been deposited at the NCBI Sequence Read Archive (http://www.ncbi.nlm.nih.gov/sra/) with the accession numbers SRR5786557 and SRR5786559 to SRR5786562.

### Mineralogical analyses

#### X-Ray Diffraction (XRD) crystallography

XRD was used for mineral phase identification by centrifugation of sample aliquots and coating wet pellets onto a XRD glass slide which was left to dry anaerobically. Samples were analyzed in an anaerobic holder fitted with an internal knife-edge on a Bruker D8 Advance, fitted with a Gobel Mirror and a Lynxeye detector. Cu Kalpha1 X-rays were used from 5–70 degrees with step size 0.02 degrees and count time of 0.5 sec per step. Eva v14 was used to match crystal patterns to standards from the ICDD (International Centre for Diffraction Data) database and a semi-quantitative analysis of relative crystallite proportions was achieved with Topas v4.2 software.

#### Transmission Electron Microscopy (TEM) and Environmental Scanning Electron Microscopy (ESEM)

TEM and ESEM were used to characterize biominerals formed during ISA degradation and to trace the fate of Ni in the solid phase. For TEM analysis, samples were centrifuged and the pellet was added to a TEM copper grid with a holey carbon film coating and let to dry anaerobically. Samples were viewed over a range of magnifications (5,000–200,000x) on a Philips FEI Technai T20 (200 kV LaB6) instrument fitted with an Oxford Instruments X-Max 80 mm^2^ SDD EDS system operating Aztec software, and a Gatan Orius SC200 CCD camera operating GMS 2 software. For ESEM imaging sample aliquots were washed in a bicarbonate buffer and dried anaerobically on an aluminum pin stub (Zeiss, Ø12.7 diameter top) before coating with carbon to enhance conductivity. The instrument used was a FEI XL30 ESEM-Field Emission Gun (ESEM-FEG) operating at 15 kV in high vacuum mode (10^−5^ to 10^−6^ mbar) secondary electron (SE) mode fitted with an EDAX Gemini EDS system for elemental analysis.

## Electronic supplementary material

Supplementary informartion

## References

[1] DECC. *Implementing Geological Disposal; A framework for the long-term management of higher activity radioactive waste*. (2014).

[2] Nuclear Decommissioning Authority & Department of Energy & Climate Change. *The 2013 UK Radioactive waste inventory: Radioactive waste composition*. (Nuclear Decommissioning Authority 2014, 2014).

[3] Whistler RL, BeMiller JN (1958). Alkaline degradation of polysaccharides. Adv. Carbohydr. Chem..

[4] van Loon, L. R. & Glaus, M. A. Technical Report 97-04: Experimental and theoretical studies on alkaline degradation of cellulose and its impact on the sorption of radionuclides. 155 (1998).

[5] Glaus MA, van Loon LR, Achatz S, Chodura A, Fischer K (1999). Degradation of cellulosic materials under the alkaline conditions of a cementitious repository for low and intermediate level radioactive waste: Part I: Identification of degradation products. Anal. Chim. Acta.

[6] Knill CJ, Kennedy JF (2003). Degradation of cellulose under alkaline conditions. Carbohydr. Polym..

[7] Pavasars I, Hagberg J, Borén H, Allard B (2003). Alkaline degradation of cellulose: Mechanisms and kinetics. J. Polym. Environ..

[8] Glaus MA, van Loon LR (2008). Degradation of Cellulose under Alkaline Conditions: New Insights from a 12 Years Degradation Study. Environ. Sci. Technol..

[9] Bradbury, M. H. & Sarott, F.-A. *Sorption Databases for the Cementitious Near-Field of a L/ILW Repository for Performance Assessment*. (1995).

[10] Loon LR, Glaus MA, Stallone S, Laube A, van (1997). Sorption of isosaccharinic acid, a cellulose degradation product, on cement. Res. Commun..

[11] Baker, S., Manning, M. C. & Williams, S. J. *The effect of cellulose degradation products on the solubility of nickel, tin and neptunium under cementitious repository condition*s. *SERCO/ERRA-0459 AEAT/R/NS/056*5 (2003).

[12] Platt JA (1997). Corrosion behavior of 2205 duplex stainless steel. Am. J. Orthod. Dentofac. Orthop..

[13] Nuclear Decommissioning Authority. *Geological Disposal: Radionuclide behaviour status report December 2010*. (Nuclear Decommissioning Authority 2010, 2010).

[14] Nuclear Decommissioning Authority & Department of Energy & Climate. *The 2013 UK radioactive waste inventory: Radioactivity content of wastes*. (Nuclear Decommissioning Authority 2014, 2014).

[15] Scheidegger AM, Wieland E, Scheinost AC, Dähn R, Spieler P (2000). Spectroscopic evidence for the formation of layered Ni-Al double hydroxides in cement. Environ. Sci. Technol..

[16] Warwick P, Evans N, Hall T, Vines S (2003). Complexation of Ni(II) by α-isosaccharinic acid and gluconic acid from pH 7 to pH 13. Radiochim. Acta.

[17] Francis AJ, Dodge CJ, Lu F, Halada GP, Clayton CR (1994). XPS and XANES studies of uranium reduction by *Clostridium* sp. Environ. Sci. Technol..

[18] Francis AJ, Geeta J-TA, Dodge CJ (1996). Biodegradation of nickel−Citrate and modulation of nickel toxicity by iron. Environ. Sci. Technol..

[19] White C, Shaman AK, Gadd GM (1998). An integrated microbial process for the bioremediation of soil contaminated with toxic metals. Nat. Biotechnol..

[20] Field EK (2010). Application of molecular techniques to elucidate the influence of cellulosic waste on the bacterial community structure at a simulated low-level-radioactive-waste site. Appl. Environ. Microbiol..

[21] Lloyd, J. R. & Renshaw, J. C. In Metal ions in Biological Systems (eds Sigel, A., Sigel, H. & Sigel, R. K.) 43, 205–240 (CRC Press, 2005).

[22] Macaskie LE (1991). The application of biotechnology to the treatment of wastes produced from the nuclear fuel cycle: Biodegradation and bioaccumulation as a means of treating radionuclide-containing streams. Crit. Rev. Biotechnol..

[23] Lloyd, J. R. & Macaskie, L. E. In *Radioactivity in the Environment* **2**, 313–342 (2002).

[24] Lloyd, J. R. & Macaskie, L. E. In Environmental Microbe-Metal Interactions (ed. Lovley, D.) 277–327, 10.1128/9781555818098, ch13 (American Society of Microbiology, 2000).

[25] Bonthrone KM, Basnakova G, Lin F, Macaskie LE (1996). Bioaccumulation of nickel by intercalation into polycrystalline hydrogen uranyl phosphate deposited via an enzymatic mechanism. Nat. Biotechnol..

[26] Basnakova G, Macaskie LE (1996). Bioaccumulation of nickel by microbially-enhanced chemisorption into polycrystalline hydrogen uranyl phosphate. Biotechnol. Lett..

[27] Basnakova G, Macaskie LE (1997). Microbially enhanced chemisorption of nickel into biologically synthesized hydrogen uranyl phosphate: A novel system for the removal and recovery of metals from aqueous solutions. Biotechnol. Bioeng..

[28] Francis AJ, Dodge CJ, Gillow JB (1992). Biodegradation of metal citrate complexes and implications for toxic-metal mobility. Nature.

[29] Joshi-Tope, G. & Francis, A. J. Mechanisms of biodegradation of metal-citrate complexes by Pseudomonas fluorescens. *J. Bacteriol*. **177**, (1995). 10.1128/jb.177.8.1989-1993.1995 PMC176840 7721690

[30] Bassil NM, Bryan N, Lloyd JR (2015). Microbial degradation of isosaccharinic acid at high pH. ISME J..

[31] Bassil, N. M., Bewsher, A. D., Thompson, O. R. & Lloyd, J. R. Microbial degradation of cellulosic material under intermediate-level waste simulated conditions. *Mineral. Mag*. **79**, (2015).

[32] Rout SP (2015). Anoxic biodegradation of isosaccharinic acids at alkaline pH by natural microbial communities. PLoS One.

[33] Charles CJ (2015). The enrichment of an alkaliphilic biofilm consortia capable of the anaerobic degradation of isosaccharinic acid from cellulosic materials incubated within an anthropogenic, hyperalkaline environment. FEMS Microbiol. Ecol..

[34] Kuippers G, Bassil NM, Boothman C, Bryan N, Lloyd JR (2015). Microbial degradation of isosaccharinic acid under conditions representative for the far field of radioactive waste disposal facilities. Mineral. Mag..

[35] Rout SP (2015). Evidence of the generation of isosaccharinic acids and their subsequent degradation by local microbial consortia within hyper-alkaline contaminated soils, with relevance to intermediate level radioactive waste disposal. PLoS One.

[36] Almond M, Belton D, Humphreys PN, Laws AP (2016). A study of the metal binding capacity of saccharinic acids formed during the alkali catalysed decomposition of cellulosic materials: nickel complexation by glucoisosaccharinic acids and xyloisosaccharinic acids. Carbohydr. Res..

[37] Howell JR, Donahoe RJ, Roden EE, Ferris FG (1998). Effects of microbial iron oxide reduction on pH and alkalinity in anaerobic bicarbonate-buffered media: implications for metal mobility. Mineral. Mag..

[38] Waychunas GA, Kim CS, Banfield JF (2005). Nanoparticulate iron oxide minerals in soils and sediments: Unique properties and contaminant scavenging mechanisms. J. Nanoparticle Res..

[39] Filip J (2011). Mechanisms and efficiency of the simultaneous removal of metals and cyanides by using ferrate(VI): Crucial roles of nanocrystalline Iron(III) oxyhydroxides and metal carbonates. Chem. - A Eur. J..

[40] Holan ZR, Volesky B (1994). Biosorption of lead and nickel by biomass of marine algae. Biotechnol. Bioeng..

[41] Tsezos M, Remoudaki E, Angelatou V (1995). A systematic study on equilibrium and kinetics of biosorptive accumulation. The case of Ag and Ni. Int. Biodeterior. Biodegradation.

[42] Berg Miller ME (2009). Diversity and strain specificity of plant cell wall degrading enzymes revealed by the draft genome of ruminococcus flavefaciens FD-1. PLoS One.

[43] Byrne-Bailey KG, Coates JD (2012). Complete genome sequence of the anaerobic perchlorate-reducing bacterium Azospira suillum strain PS. J. Bacteriol..

[44] Su X-L (2014). An anaerobic hydrogen-producing bacterium in the family Rikenellaceae isolated from a reed swamp. IInternational J. Syst. Evol. Microbiol..

[45] Eggleton RA, Fitzpatrick RW (1988). New data and a revised structural model for ferrihydrite. Clays Clay Miner..

[46] Drits VA, Sakharov BA, Salyn AL, Manceau A (1993). Structural model for ferrihydrite. Clay Miner..

[47] Janney DE, Cowley JM, Buseck PR (2000). Transmission electron microscopy of synthetic 2- and 6-line Fe(III) oxyhydroxide. Clays Clay Miner..

[48] Guo H, Barnard AS (2013). Naturally occurring iron oxide nanoparticles: morphology, surface chemistry and environmental stability. J. Mater. Chem. A.

[49] Bartelmehs, K. & Downs, B. R040034. (1998). Available at, http://rruff.info/repository/sample_child_record_powder/by_minerals/Siderite__R040034-1__Powder__Cell_Refinement_Output_Data__3109.txt. (Accessed: 16th February 2017)

[50] Kim J, Dong H, Seabaugh J, Newell SW, Eberl DD (2004). Role of Microbes in the Smectite-to-Illite Reaction. Science (80-.)..

[51] Evans HT (1964). Valleriite and the new iron sulfide mackinawite. Mineral. Petrol..

[52] Lennie AR, Redfern SAT, Schofield PF, Vaughan DJ (1995). Synthesis and Rietveld crystal structure refinement of mackinawite, tetragonal FeS. Mineral. Mag..

[53] Wolthers M, Van der Gaast SJ, Rickard D (2003). The structure of disordered mackinawite. Am. Mineral..

[54] Ohfuji, H. & Rickard, D. High resolution transmission electron microscopic study of synthetic nanocrystalline mackinawite. *Earth Planet. Sci. Lett*. 227–233, 10.1016/j.epsl.2005.10.006 (2006).

[55] Gramp JP, Bigham JM, Jones FS, Tuovinen OH (2010). Formation of Fe-sulfides in cultures of sulfate-reducing bacteria. J. Hazard. Mater..

[56] Lentini CJ, Wankel SD, Hansel CM (2012). Enriched iron(III)-reducing bacterial communities are shaped by carbon substrate and iron oxide mineralogy. Front. Microbiol..

[57] Lovley DR, Phillips EJ (1988). Novel mode of microbial energy metabolism: organic carbon oxidation coupled to dissimilatory reduction of iron or manganese. Appl. Environ. Microbiol..

[58] Lovley DR, Woodward J, Chapelle F, Nature (1994). Stimulated anoxic biodegradation of aromatic hydrocarbons using Fe(III) ligands. Nature.

[59] Dobbin PS, Powell AK, McEwan AG, Richardson DJ (1995). The influence of chelating agents upon the dissimilatory reduction of Fe(III) by Shewanella putrefaciens. BioMetals.

[60] Dobbin PS (1996). The influence of chelating agents upon the dissimilatory reduction of Fe(III) byShewanella putrefaciens. Part 2. Oxo-and hydroxo-bridged polynuclear Fe(III) complexes. BioMetals.

[61] Islam FS (2005). Interactions between the Fe(III)-reducing bacterium Geobacter sulfurreducens and arsenate, and capture of the metalloid by biogenic Fe(II). Appl. Environ. Microbiol..

[62] McBeth, J. M. *et al*. Redox interactions of technetium with iron-bearing minerals. *Mineral. Mag*. **75**, (2011).

[63] Bard, A. J., Parsons, R. & Jordan, J. *Standard potentials in aqueous solution*. (M. Dekker, 1985).

[64] Fredrickson JK (2001). Biotransformation of Ni-substituted hydrous ferric oxide by an Fe(III)-reducing bacterium. Environ. Sci. Technol..

[65] Roh Y (2003). Biogeochemical and environmental factors in Fe biomineralization: Magnetite and siderite formation. Clays Clay Miner..

[66] Lloyd JR, Sole VA, Van Praagh CV, Lovley DR (2000). Direct and Fe(II)-mediated reduction of technetium by Fe(III)-reducing bacteria. Appl. Environ. Microbiol..

[67] Thorpe CL (2014). The interactions of strontium and technetium with Fe(II) bearing biominerals: Implications for bioremediation of radioactively contaminated land. Appl. Geochemistry.

[68] Law GTW (2010). Geomicrobiological Redox Cycling of the Transuranic Element Neptunium. Environ. Sci. Technol..

[69] Veeramani H (2011). Products of abiotic U(VI) reduction by biogenic magnetite and vivianite. Geochim. Cosmochim. Acta.

[70] Takeno S, Zoka H, Nihara T (1970). Metastable cubic iron sulfide-with special reference to mackinawite. Am. Mineral..

[71] Schoonen MAA, Barnes HL (1991). Reactions forming pyrite and marcasite from solution: I. nucleation of FeS_2_ below 100 °C. Geochim. Cosmochim. Acta.

[72] Morse JW, Arakaki T (1993). Adsorption and coprecipitation of divalent metals with mackinawite (FeS). Geochim. Cosmochim. Acta.

[73] Clark AH (1970). Nickelian mackinawite from Vlakfontein, Transfaal: A discussion. Mineral. Notes.

[74] Kwon KD, Refson K, Sposito G (2015). Transition metal incorporation into mackinawite (tetragonal FeS). Am. Mineral..

[75] Gramp JP, Bigham JM, Sasaki K, Tuovinen OH (2007). Formation of Ni- and Zn-Sulfides in Cultures of Sulfate-Reducing Bacteria. Geomicrobiol. J..

[76] Nielsen FH, Ollerich DA (1974). Nickel: a new essential trace element. Fed. Proc..

[77] Babich H, Stotzky G (1983). Further studies on environmental factors that modify the toxicity of nickel to microbes. Regul. Toxicol. Pharmacol..

[78] Pümpel T, Macaskie LE, Finlay JA, Diels L, Tsezos M (2003). Nickel removal from nickel plating waste water using a biologically active moving-bed sand filter. BioMetals.

[79] Madigan, M. T., *et al**Brock biology of microorganisms*. (Pearson Education Limited, 2015).

[80] Poulson SR, Colberg PJS, Drever JI (1997). Toxicity of heavy metals (Ni, Zn) to *Desulfovibrio desulfuricans*. Geomicrobiol. J..

[81] Ruggiero CE (2005). Actinide and metal toxicity to prospective bioremediation bacteria. Environ. Microbiol..

[82] Metcalfe R (2007). Characteristics of deep groundwater flow in a basin marginal setting at Sellafield, Northwest England: 36Cl and halide evidence. Appl. Geochemistry.

[83] Rizoulis A, Steele HM, Morris K, Lloyd JR (2012). The potential impact of anaerobic microbial metabolism during the geological disposal of intermediate-level waste. Mineral. Mag..

[84] Carvalho WA, Vignado C, Fontana J (2008). Ni(II) removal from aqueous effluents by silylated clays. J. Hazard. Mater..

[85] Suresh K, Borah M, Jatty SK (2009). Adsorption of nickel by bentonite clays; A comparative study. J. Envionmental Sci. Eng..

[86] Williamson AJ (2013). Microbial reduction of Fe(III) under alkaline conditions relevant to geological disposal. Appl. Environ. Microbiol..

[87] Lovley DR, Greening RC, Ferry JG (1984). Rapidly growing rumen methanogenic organism that synthesizes coenzyme M and has a high affinity for formate. Appl. Environ. Microbiol..

[88] Vercammen K (1999). Complexation of calcium by alpha-isosaccharinic acid under alkaline conditions. Acta Chem. Scand..

[89] Stookey LL (1970). Ferrozine-a new spectrophotometric reagent for iron. Anal. Chem..

[90] Lovley DR, Phillips EJ (1986). Organic matter mineralization with reduction of ferric iron in anaerobic sediments. Appl. Environ. Microbiol..

[91] Caporaso JG (2011). Global patterns of 16S rRNA diversity at a depth of millions of sequences per sample. Proc. Natl. Acad. Sci. USA.

[92] Caporaso JG (2012). Ultra-high-throughput microbial community analysis on the Illumina HiSeq and MiSeq platforms. ISME J.

[93] Kozich JJ, Westcott SL, Baxter NT, Highlander SK, Schloss PD (2013). Development of a dual-index sequencing strategy and curation pipeline for analyzing amplicon sequence data on the MiSeq Illumina sequencing platform. Appl. Environ. Microbiol..

[94] Martin M (2011). Cutadapt removes adapter sequences from high-throughput sequencing reads. EMBnet.journal.

[95] Andrews, S. FastQC A quality control tool for high throughput sequence data (2010).

[96] Joshi, N. A. & Fass, J. N. Sickle: A sliding-window, adaptive, quality-based trimming tool for FastQ files (2011).

[97] Nurk, S. *et al*. In *Research in Computational Molecular Biology: 17th Annual International Conference, RECOMB 2013, Beijing, China, April 7*–*10, 2013* (eds Deng, M., Jiang, R., Sun, F. & Zhang, X.) 158–170, 10.1007/978-3-642-37195-0_13 (Springer, Berlin, Heidelberg, 2013).

[98] Masella AP, Bartram AK, Truszkowski JM, Brown DG, Neufeld JD (2012). PANDAseq: paired-end assembler for illumina sequences. BMC Bioinformatics.

[99] Haas BJ (2011). Chimeric 16S rRNA sequence formation and detection in Sanger and 454-pyrosequenced PCR amplicons. Genome Res..

[100] Edgar RC (2013). UPARSE: highly accurate OTU sequences from microbial amplicon reads. Nat. Methods.

[101] Edgar RC (2010). Search and clustering orders of magnitude faster than BLAST. Bioinformatics.

[102] Wang Q, Garrity GM, Tiedje JM, Cole JR (2007). Naive Bayesian classifier for rapid assignment of rRNA sequences into the new bacterial taxonomy. Appl. Environ. Microbiol..

[103] Caporaso, G. J. *et al*. QIIME allows analysis of high-throughput community sequencing data. *Nat. Publ. Gr*. **7**, (2010). 10.1038/nmeth.f.303 PMC3156573 20383131

