# Supplementary material for: The biogeochemical fate of nickel during microbial ISA degradation; implications for nuclear waste disposal
Source: Sci Rep. 2018 Jun 8;8:8753. doi: 10.1038/s41598-018-26963-8 (PMC5993814; doi:10.1038/s41598-018-26963-8)
Supplement: Supplementary file 1 — Supplementary informartion [file 41598_2018_26963_MOESM1_ESM.docx]

Supplementary information

**Title: The biogeochemical fate of nickel during microbial ISA degradation; implications for nuclear waste disposal**

Author list: Gina Kuippers, Christopher Boothman, Heath Bagshaw, Michael Ward, Rebecca Beard, Nicholas Bryan, Jonathan R. Lloyd

Figure-S1. Pre-test for Ni solubility in freshwater minimal medium (FWM) after a week of incubation in the dark at 20°C. Tests at 0.1 mM and 1 mM Ni concentrations include (i) FWM only; (ii) Fe(III), (iii) ISA, (iv) Fe(III) and ISA.

Figure-S2. Sulfate **concentration in Ni-ISA biostimulation, Fe(III)-reducing experiment. Symbols are: (****) sterile control, (****) 0 mM Ni, and (**
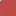
**) 0.1 mM Ni.**

Figure-S3. **α-rarefaction curves 16S rRNA gene sequencing data from Fe(III)-reducing Ni-ISA degrading microbially active enrichments showing maximum of distinct species per sample.**


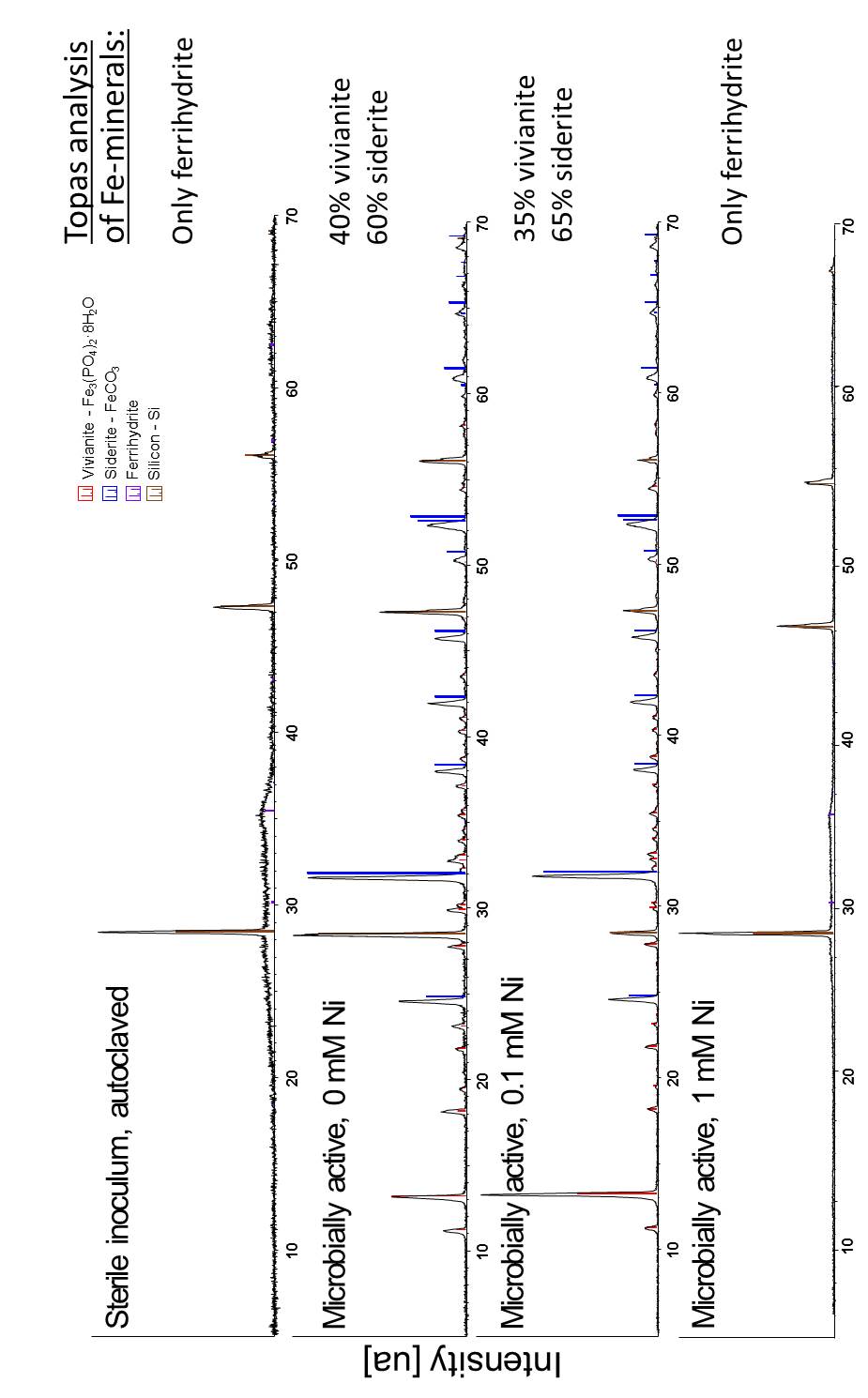
Figure-S4. XRD patterns of Fe(III)-reducing Ni-ISA degrading enrichmentshowing: A) Sterile control, 1 mM Ni; microbially active with B) 0 mM Ni, C) 0.1 mM Ni, or D) 1 mM Ni; E) Abiotic control without microbial inoculum and with 1 mM Ni.

Figure-S5. **TEM mapping of a precipitate at the end of the Fe(III)-reducing Ni-ISA degrading enrichment at 0.1 mM Ni. Detected elements are displayed in maps showing O, Fe, P, Ca, Ni and S.**


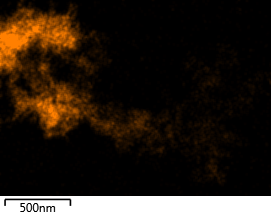

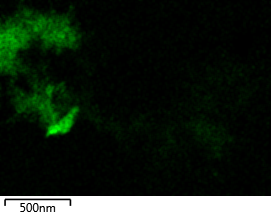

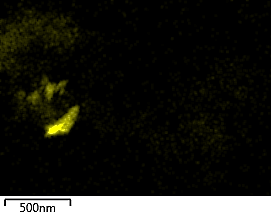

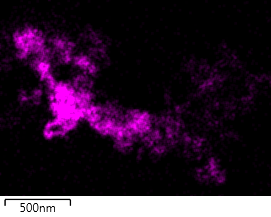

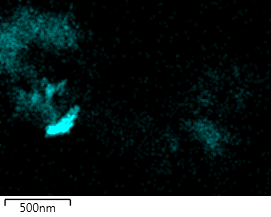

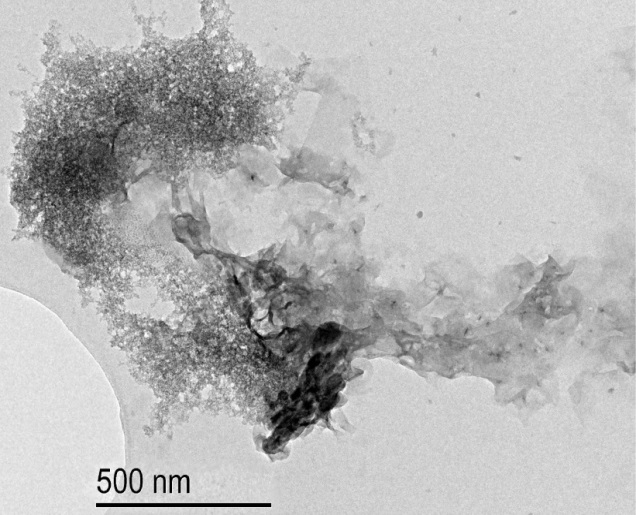

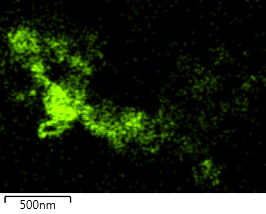


**O**

**Fe**

**Ni**

**P**

**Ca**

**S**

Figure-S6. PHREEQC modelling output file showing Ni-ISA biostimulation, Fe(III)-reducing experiment with 0.1 mM Ni at incubation start (before bio-reduction). The system is in equilibrium with ferrihydrite. Saturation indices show that vivianite, siderite and apatite will precipitate (positive value), but no Ni-containing form is expected to precipitate.


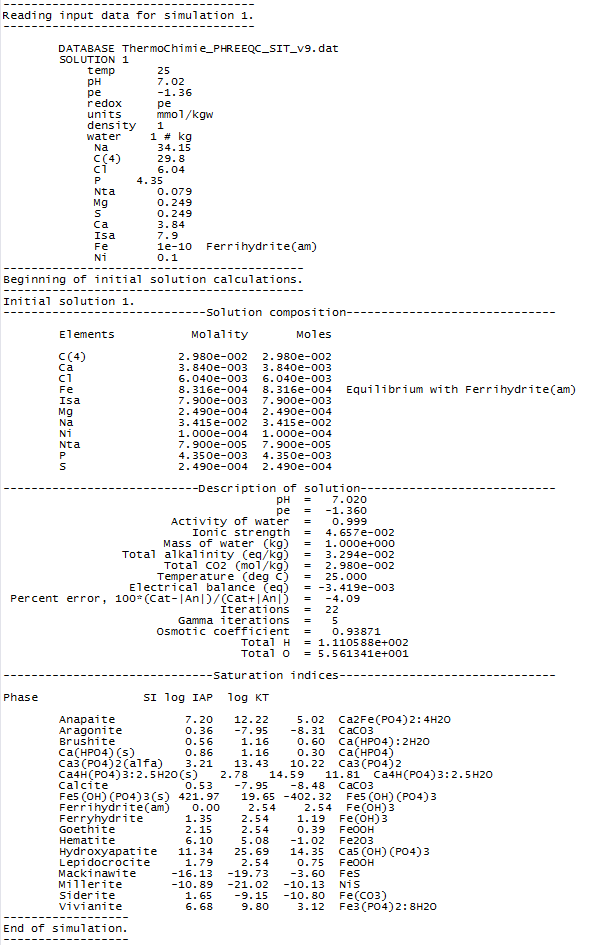


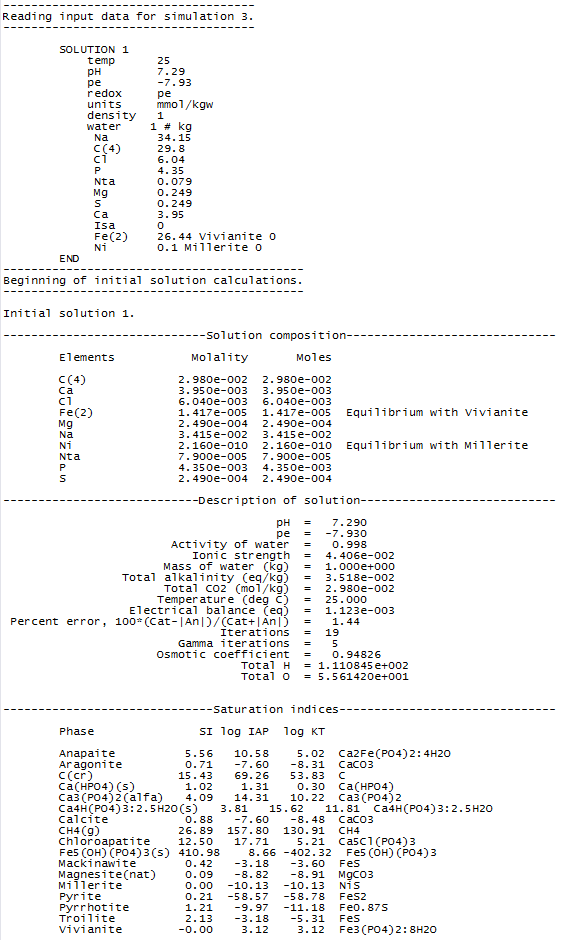
Figure-S7. PHREEQC modelling output file showing Ni-ISA biostimulation, Fe(III)-reducing experiment with 0.1 mM Ni when bioreduced (negative pe). The system is in equilibrium with vivianite and millerite. Saturation indices show supersaturation with mackinawite. Thus the simulation indicates millerite and mackinawite will form, which will under natural conditions be a mixed form.
